# Supplementary material for: Scalable optimal Bayesian classification of single-cell trajectories under regulatory model uncertainty
Source: BMC Genomics. 2019 Jun 13;20(Suppl 6):435. doi: 10.1186/s12864-019-5720-3 (PMC6561847; doi:10.1186/s12864-019-5720-3)
Supplement: Supplementary file 1 — This additional file contains the additional experiment results. (PDF 229 kb) [file 12864_2019_5720_MOESM1_ESM.pdf]

Scalable Optimal Bayesian Classification of Single-Cell Trajectories under Regulatory Model Uncertainty

Ehsan Hajiramezanali, Mahdi Imani, Ulisses Braga-Neto, Xiaoning Qian, and Edward R. Dougherty

This document contains the supplementary materials for the paper "Scalable Optimal Bayesian Classification of Single-Cell Trajectories under Regulatory Model Uncertainty".

Additional Experimental Results

In addition to the results for testing different Gaussian noise levels in the main text, we provide more detailed experimental results for different noise distributions as gene expression noise based on the gene regulatory network (GRN) model can also be Poisson or Negative Binomial (NB). Specifically, Let $\mathbf{Y}_k$ denote the expression value at time k. To compare different noise models, the observation models as discussed in the main text are set to $(\mathbf{Y}_k|\mathbf{X}_k, \boldsymbol{\lambda}, D) \sim \mathcal{N}(\boldsymbol{\lambda} + D\mathbf{X}_k, \sigma^2 I_n)$, $(\mathbf{Y}_k|\mathbf{X}_k, \boldsymbol{\lambda}, D) \sim \mathrm{NB}(\boldsymbol{r}_{\mathrm{NB}}, \boldsymbol{p}_{\mathrm{NB}})$, and $(\mathbf{Y}_k|\mathbf{X}_k, \boldsymbol{\lambda}, D) \sim \mathrm{Poisson}(\boldsymbol{\lambda} + D\mathbf{X}_k)$ for Gaussian, Negative Binomial, and Poisson noise distributions, respectively. For NB parameters, $\boldsymbol{r}_{\mathrm{NB}} = \frac{(\boldsymbol{\lambda} + D\mathbf{X}_k)^2}{\sigma^2 I_n - (\boldsymbol{\lambda} + D\mathbf{X}_k)}$ and $\boldsymbol{p}_{\mathrm{NB}} = \frac{\sigma^2 I_n - (\boldsymbol{\lambda} + D\mathbf{X}_k)}{\sigma^2 I_n}$. For the sake of simplicity, we assume the gene-expression parameters to be the same for all genes and $\boldsymbol{\lambda} = [\lambda, ..., \lambda]^T$ and $D = \mathrm{Diag}(\delta, ..., \delta)$, with $\lambda = 10$, $\delta = 30$, and $\sigma = 20$. Figure S1 shows that the proposed particle filter based method can work consistently well in different noise distributions. This is mostly due to the generalizability of particle filter.

Regarding the performance of auxiliary particle filter (APF) in high process noise, we compared it with the plain Sequential Important Resampling (SIR) as well. As Figure S2 shows, the performance is similar especially when there is enough time points. When the number of time points is low, the SIR based particle filter has the worse performance. Based on this comparison, we decided to focus on the APF method. The superior performance is mostly due to the more efficient resampling step of APF which is based on the predicted modes of particles in the next time step, while the random resampling process of SIR algorithm poses the significant errors during the estimation process.

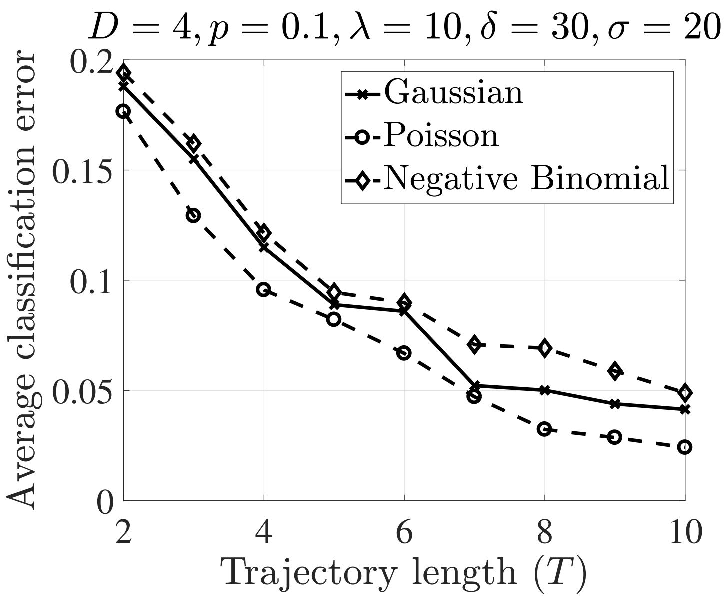

Figure S1: Trajectory-based classification results for different noise distributions.

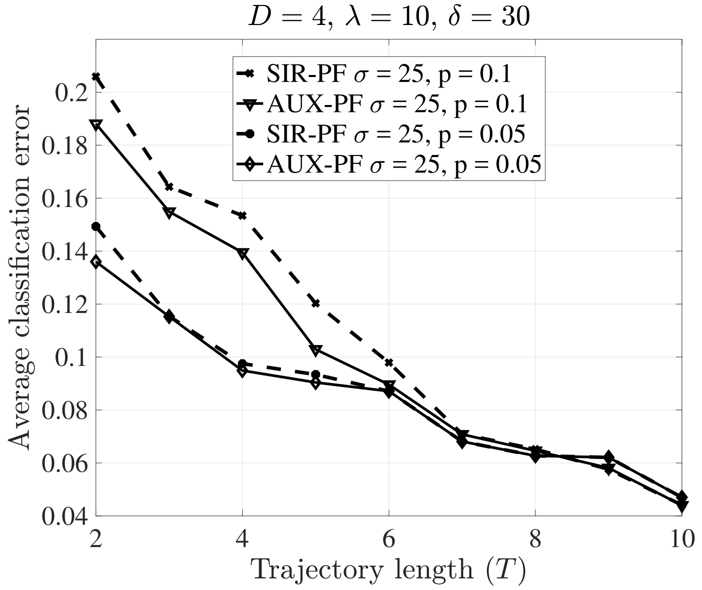

Figure S2: Comparison between SIR-based and auxiliary particle filters for different p in high noise scenario.
